# Public awareness of risk factors for cancer among the Japanese general population: A population-based survey

**DOI:** 10.1186/1471-2458-6-2

**Published:** 2006-01-10

**Authors:** Manami Inoue, Motoki Iwasaki, Tetsuya Otani, Shizuka Sasazuki, Shoichiro Tsugane

**Affiliations:** 1 Epidemiology and Prevention Division, Research Center for Cancer Prevention and Screening, National Cancer Center, Tokyo, Japan

## Abstract

**Background:**

The present study aimed to provide information on awareness of the attributable fraction of cancer causes among the Japanese general population.

**Methods:**

A nationwide representative sample of 2,000 Japanese aged 20 or older was asked about their perception and level of concern about various environmental and genetic risk factors in relation to cancer prevention, as a part of an Omnibus Survey. Interviews were conducted with 1,355 subjects (609 men and 746 women).

**Results:**

Among 12 risk factor candidates, the attributable fraction of cancer-causing viral and bacterial infection was considered highest (51%), followed by that of tobacco smoking (43%), stress (39%), and endocrine-disrupting chemicals (37%). On the other hand, the attributable fractions of cancer by charred fish and meat (21%) and alcohol drinking (22%) were considered low compared with other risk factor candidates. For most risk factors, attributable fraction responses were higher in women than in men. As a whole, the subjects tended to respond with higher values than those estimated by epidemiologic evidence in the West. The attributable fraction of cancer speculated to be genetically determined was 32%, while 36% of cancer was considered preventable by improving lifestyle.

**Conclusion:**

Our results suggest that awareness of the attributable fraction of cancer causes in the Japanese general population tends to be dominated by cancer-causing infection, occupational exposure, air pollution and food additives rather than major lifestyle factors such as diet.

## Background

In Japan, cancer has been recognized as a major component of the overall pattern of disease for decades. Thus, the importance of cancer prevention by lifestyle modification should now be strongly acknowledged.

Internationally, several studies have estimated the proportion of total cancer deaths attributable to various risk factors based on epidemiologic evidence [1,2], and various international guidelines and recommendations derived from these have appeared [3-6]. Not surprisingly, domestic guidelines and recommendations for cancer prevention in Japan such as the 'Twelve recommendations for cancer prevention [7]' and 'Healthy People Japan 21 [8]' have been significantly influenced by these reports.

Public awareness of risk factors in relation to cancer prevention has been surveyed in only a few countries [9,10], and results have demonstrated poor awareness. Other studies focusing on specific cancers only have also appeared [11-14]. However, none of these studies quantitatively evaluated public awareness of the attributable fraction of individual risk factors.

In Japan, it appears that most people are aware of the major risk factors of cancer. Although we are unaware of any published evidence, however, public knowledge and information on cancer prevention now seems influenced largely by the mass media and other sources, rather than by information provided directly by health professionals, resulting in a distorted picture of causation. Cancer control policy therefore urgently requires a clarification of the discrepancies which now exist between ideal levels of public concern about risk factors and the current reality, particularly public health policy makers in their formulation of cancer control measures. To address this need, the present study was designed to provide information on awareness of the attributable fraction of cancer causes among the Japanese general population. Since we are interested in quantitatively estimating the awareness of preventability, we placed special emphasis on gauging awareness by attributable fraction of cancer.

## Methods

The study was conducted as a part of an omnibus survey in December, 2003, by commission to a polling agency. The omnibus survey is a monthly multipurpose cross-sectional survey which includes public opinion research, social research, scientific research, market research, and others. Using a stratified two-stage sampling method, a total of 2,000 people aged 20 or older were randomly selected as study subjects, from 160 districts selected from area units representing 12 geographical blocks (Hokkaido, Tohoku, Kanto, Keihin, Koshinetu, Hokuriku, Tokai, Kinki, Hanshin, Chugoku, Shikoku, Kyushu) and 3 types of city scale (14 metropolises, other cities, towns and villages) in proportion to the population distribution as at March 2002. After an initial visit to obtain oral informed consent and schedule a visit for the interview, the survey was conducted by face-to-face interview using trained interviewers in each district. The omnibus survey does not collect any personally identifiable information such as name, date of birth or address details at interview. For the present report, we obtained the electronic data file for the relevant interview component, with no personal identifiers. Ethical approval was not applicable to the present study under the Japanese ethical guidelines for epidemiologic studies, which comply with the declaration of Helsinki.

Among the 2,000 people selected for survey (977 men, 1,023 women), interviews were successfully obtained from 1,355 (67.8%). The remaining 645 did not respond because of change of address after sampling (n = 29), absence from home in the survey period (n = 295), refusal to participate (n = 303), and other undetermined reasons (n = 18).

The questionnaire of this survey comprised questions on the awareness of various environmental and genetic risk factors in relation to cancer prevention by enquiring about the attributable fraction of cancer. Fractions were: 1) 12 risk factor candidates, namely alcoholic beverages, unbalanced diet, use of food additives and pesticide chemicals, charred fish and meat, tobacco smoking, obesity, physical inactivity, endocrine-disrupting chemicals, air pollution such as diesel emissions, occupational exposure, cancer-causing viral and bacterial infection, and stress; 2) genetic factors in general; and 3) the preventable fraction of cancer occurrence by lifestyle modification [see Additional file 1].

The first question asked about the preventable fraction of cancer which would result in Japan if each factor were completely and totally eliminated, using the fine categories of <5%, 5 to <10%, 10 to <15%, 15 to <20%, 20 to <25%, 25 to <30%, 30 to <40%, 40 to <50%, 50 to <60%, 60 to <70%, 70 to <80%, 80 to <90%, and 90 to 100%. These categories were exhibited together on a pie chart. These risk factor candidates were selected with reference to previous international and domestic recommendations and guidelines [1-8]. The second question asked about the fraction of cancer genetically predetermined using the same categories as the first, while the third asked about the preventable fraction of cancer by modification of lifestyle using estimation of an actual percent value. In addition to these questions, subjects were also asked about their smoking and drinking practices, and occupational and educational status.

Mean values of the attributable fractions were calculated for each risk factor of cancer and compared by demographic and habitual smoking and drinking status. For analyses, the mid-values of each category were assigned for categorical variables. All analyses were performed using Stata statistical software, S/E Version 8 [15].

## Results

A total of 1,355 (67.8%) subjects responded to the survey, with a higher response rate in women (72.9%) than in men (62.3%). Response rate was lower in the 20s age strata than in the other age groups, but no trend to an increase in response rate with increasing age was observed. Overall, no significant difference in area and age distribution was seen between the sampled population and survey respondents. Response rate tended to be lower among subjects who reside in the Kanto region and in cities other than the 14 metropolises than among other subjects (Table 1).

Characteristics of the 1,355 respondents (609 men, 746 women) are presented in Table 2. The proportion of current smokers was 44% in men and 15% in women, and decreased with age in both genders. In female subjects aged in their 20s, 26% currently smoke and 49% drink alcohol beverages at least 4 times a week.

Awareness of the attributable fraction of cancer causes among the Japanese general population is presented in Table 3. Among the 12 risk factor candidates, the attributable fraction was considered highest for cancer-causing viral and bacterial infection (51.3%), followed by tobacco smoking (43.0%), stress (39.0%), and endocrine-disrupting chemicals (37.1%). In contrast, the attributable fraction of charred fish and meat (21.4%) and alcohol drinking (21.7%) were considered low compared with other risk factor candidates. The attributable fraction of other risk factor candidates such as occupational exposure, air pollution, food additives and pesticides, unbalanced diet, obesity and physical activity ranked between the high and low fractions. The attributable fraction responses tended to be higher in women than in men, and were increased among inhabitants of larger cities and in homemakers and decreased in those engaged in agriculture, forestry and fisheries. In contrast, risk factor candidate rankings were similar by gender, age group, city scale, and educational and occupational status. In men, those who neither smoke nor drink tended to consider the preventive fraction of the risk factors higher than those who both smoke and drink, whereas in women, the former subjects considered the values lower than the latter.

The speculated fraction of cancer which is genetically determined was 31.5% as an average (Table 3). This fraction was higher in current heavy smokers and former drinkers, and lower in homemakers and students. On the other hand, an average 35.5% of cancer were considered preventable by lifestyle improvement, with this ratio being higher in homemakers, former smokers, and never and former drinkers.

## Discussion

The present survey, targeted at the Japanese general population, showed that the attributable fraction of cancer among Japanese tended to be higher for cancer-causing infection, occupational exposure, air pollution and food additives than major lifestyle factors such as dietary factors. In addition, the attributable fraction of cancer estimated by the Japanese general population was higher than that derived from epidemiologic evidence in the West, which is frequently quoted as 30% for tobacco smoking and 30% for food as a whole [1,2].

Some of the major cancers in Japan, including gastric and liver cancers, are known to be related to cancer-causing viral and bacterial infection, and a higher level of concern about such infection among Japanese than in Western populations would therefore be understandable [9]. Notwithstanding the validity of such concern, however, the high level of concern for infection, as well as for endocrine-disrupting chemicals, identified in the present survey was most likely due to the severe acute respiratory syndrome (SARS) epidemic which occurred just prior to it, as well as a focus on endocrine-disrupting chemicals in the Japanese mass media in recent years. Both of these resulted in a surge of interest in these issues, even if their relationship with cancer is less likely.

Likewise, a high level of concern for tobacco smoking was also observed, in spite of a relatively dull reduction in the rate of male current smokers in past decades compared with the U.S. This was probably due to recent enactment of the Health Promotion Law, which curbs passive smoking in public spaces.

Respondent estimates for attributable fractions were generally high. This may be in part due to anchoring and adjustment effects of the response categories used and the tendency of people to respond near the middle of the scale. Given that responses tended to be generally high, concern over the present results should probably be focused on rankings rather than absolute values per se. Although tobacco smoking ranked among the top factors, risk factor candidates whose actual contribution is considered to be low, such as endocrine-disrupting chemicals, occupational exposure, air pollution such as diesel emissions and the use of food additives and pesticide chemicals ranked higher than previous estimates of the attributable fraction of cancer causes [1,2]. In contrast, this should be compared with the results for unbalanced diet, which ranked at only 8th among the 12 risk factor candidates despite an actual ranking which is estimated to be as high as that for tobacco smoking. Particularly in light of findings on long-term exposure to common lifestyle factors such as diet as a cause of cancer, these results suggest that public awareness of cancer prevention is still insufficient.

We are unaware of any previous studies aimed at determining public awareness of the attributable fraction of cancer as a whole or at gauging the level the awareness of cancer prevention by attributable fraction. Accordingly, to our knowledge, this is the first attempt to discover the level of awareness for each risk factor candidate, and the questionnaire used has hence not been fully validated. In addition, as indicated above, responses to this type of cross sectional survey are subject to social conditions such as information from the mass media and other sources on disease epidemics and other putative risk factors. Thus, the results might not necessarily reflect actual public awareness. However, the study subjects were recruited from among a nationally representative random sample, and the response rate was similar to that of recent omnibus surveys in other countries [16-19]. Nevertheless, the exclusion of non-respondents may have distorted the results.

## Conclusion

In conclusion, awareness of the attributable fraction of cancer causes among the Japanese general population tended to be dominated by infection, occupational exposure, air pollution and food additives rather than dietary factors. The results of the present survey provide valuable clues and perspectives toward the formulation of relevant cancer prevention strategies in Japan.

## Competing interests

The author(s) declare that they have no competing interests.

## Authors' contributions

MIn developed the concept and design of the study, analyzed the data and wrote the paper. MIw, TO and SS participated in the design of the study, interpretation of the data and revision of the paper. ST developed the concept and design of the study and revision of the paper. All authors read and approved the final manuscript.

## Pre-publication history

The pre-publication history for this paper can be accessed here:



## Supplementary Material

Additional File 1 Questionnaire in the omnibus survey on the awareness of risk factors and prevention of cancer Click here for file

## Figures and Tables

**Table 1:** Survey response rate

	Number of samples	Number of responses	%
Total	2000	1355	67.8
Gender			
Men	977	609	62.3
Women	1023	746	72.9
Age			
20–29	284	175	61.6
30–39	351	247	70.4
40–49	369	257	69.6
50–59	411	282	68.6
60–69	349	228	65.3
70≤	236	166	70.3
Region			
Hokkaido and Tohoku	245	169	69.0
Kanto (Kanto and Keihin)	646	425	65.8
Chubu (Koshinetsu, Hokuriku and Tokai)	366	261	71.3
Kinki (Kinki and Hanshin)	325	222	68.3
Chugoku, Shikoku and Kyushu	418	278	66.5
City scale			
14 metropolises*	458	325	71.0
Other cities	1122	736	65.6
Towns and villages	420	294	70.0

* Sapporo, Sendai, Saitama, Chiba, Tokyo, Kawasaki, Yokohama, Nagoya, Kyoto, Osaka, Kobe, Hiroshima, Kitakyushu and Fukuoka.

**Table 2:** Respondent characteristics

	Total			Male			Female		
	
	Number	%	(SE)	Number	%	(SE)	Number	%	(SE)
Total	1355	100		609	44.9		746	55.1	
Educational status									
Junior high school	202	14.9	(1.0)	93	15.3	(1.5)	109	14.6	(1.3)
Senior high school	693	51.2	(1.4)	273	44.9	(2.0)	420	56.3	(1.8)
College or higher	459	33.9	(1.3)	242	39.8	(2.0)	217	29.1	(1.7)
Occupation									
Agriculture, forestry and fisheries	35	2.6	(0.4)	17	2.8	(0.7)	18	2.4	(0.6)
Labor	276	20.4	(1.1)	127	20.9	(1.6)	149	20.0	(1.5)
Service, clerk, management, others	659	48.6	(1.4)	439	72.1	(1.8)	220	29.5	(1.7)
Homemaker	351	25.9	(1.2)	0	0.0	(0.0)	351	47.1	(1.8)
Student	34	2.5	(0.4)	26	4.3	(0.8)	8	1.1	(0.4)
Habitual smoking									
Never	724	53.4	(1.4)	158	25.9	(1.8)	566	75.9	(1.6)
Former	256	18.9	(1.1)	186	30.5	(1.9)	70	9.4	(1.1)
Current (<20 cigarettes per day)	245	18.1	(1.0)	149	24.5	(1.7)	96	12.9	(1.2)
Current (≥20 cigarettes per day)	130	9.6	(0.8)	116	19.0	(1.6)	14	1.9	(0.5)
Habitual drinking									
Never	640	47.2	(1.4)	177	29.1	(1.8)	463	62.1	(1.8)
Former	34	2.5	(0.4)	19	3.1	(0.7)	15	2.0	(0.5)
Current (≤4 times per week)	423	31.2	(1.3)	209	34.3	(1.9)	214	28.7	(1.7)
Current (almost everyday)	258	19.1	(1.1)	204	33.5	(1.9)	54	7.2	(0.9)

SE: Standard error

**Table 3:** Awareness of attributable fraction of cancer causes among the Japanese general population

	Total		Men		Women	
	
	Mean %	(95%CI)	Mean %	(95%CI)	Mean %	(95%CI)
Preventable fraction of cancer (%) by eliminating:						
Cancer-causing viral and bacterial infection	51.3	(49.5- 53.0)	48.7	(46.1- 51.3)	53.4	(51.1- 55.6)
Tobacco smoking	43.0	(41.6- 44.4)	40.1	(38.1- 42.2)	45.4	(43.6- 47.3)
Stress	39.0	(37.6- 40.4)	35.8	(33.7- 37.9)	41.6	(39.7- 43.5)
Endocrine-disrupting chemicals	37.1	(35.7- 38.5)	34.9	(32.8- 37.0)	38.9	(37.1- 40.8)
Occupational exposure	36.0	(34.5- 37.4)	33.2	(31.1- 35.4)	38.2	(36.3- 40.2)
Air pollution	34.7	(33.4- 36.0)	32.5	(30.5- 34.5)	36.6	(34.7- 38.4)
Food additives and pesticides	31.4	(30.2- 32.7)	28.7	(26.9- 30.5)	33.7	(32.0- 35.4)
Unbalanced diet	28.8	(27.6- 30.0)	26.1	(24.4- 27.7)	31.0	(29.4- 32.7)
Obesity	28.2	(27.0- 29.4)	25.4	(23.6- 27.1)	30.6	(28.9- 32.3)
Physical inactivity	26.0	(24.8- 27.1)	23.8	(22.1- 25.5)	27.8	(26.2- 29.4)
Alcohol drinking	21.7	(20.7- 22.7)	19.2	(17.8- 20.6)	23.7	(22.3- 25.1)
Charred fish and meat	21.4	(20.3- 22.5)	20.1	(18.4- 21.8)	22.5	(21.0- 23.9)
						
Fraction of cancer genetically determined (%)	31.5	(30.2- 32.9)	32.0	(30.0- 34.1)	31.1	(29.4- 32.8)
Fraction of cancer preventable by improving lifestyle (%)	35.5	(34.6- 36.5)	33.7	(32.3- 35.2)	37.0	(35.7- 38.3)

CI: Confidence interval
