# Supplementary material for: Public awareness of risk factors for cancer among the Japanese general population: A population-based survey
Source: BMC Public Health. 2006 Jan 10;6:2. doi: 10.1186/1471-2458-6-2 (PMC1351169; doi:10.1186/1471-2458-6-2)
Supplement: Additional File 1 — Questionnaire in the omnibus survey on the awareness of risk factors and prevention of cancer [file 1471-2458-6-2-s1.doc]

Questionnaire in the omnibus survey on the awareness of risk factors and prevention of cancer

| Question 1: What percentage of cancer occurring in Japan do you think is preventable if the following were completely and totally eliminated?  (1) <5% (2) 5 to <10% (3) 10 to <15% (4) 15 to <20% (5) 20 to <25%  (6) 25 to <30% (7) 30 to <40% (8) 40 to <50% (9) 50 to <60% (10) 60 to <70%  (11) 70 to <80% (12) 80 to <90% (13) 90 to 100% (14) do not know   1. Consumption of alcoholic beverages 2. Unbalanced diet such as a diet low in vegetables and fruit and high in salt 3. Use of food additives and pesticide chemicals 4. Consumption of charred fish and meat 5. Tobacco smoking 6. Obesity 7. Physical inactivity 8. Endocrine-disrupting chemicals such as dioxin 9. Air pollution such as diesel emissions 10. Occupational exposure to harmful substances 11. Infection with viruses and bacteria which cause cancer 12. High stress   Question 2: What percentage of cancer do you think is genetically predetermined?  (1) <5% (2) 5 to <10% (3) 10 to <15% (4) 15 to <20% (5) 20 to <25%  (6) 25 to <30% (7) 30 to <40% (8) 40 to <50% (9) 50 to <60% (10) 60 to <70%  (11) 70 to <80% (12) 80 to <90% (13) 90 to 100% (14) do not know  Question 3: What percentage of cancer do you think is preventable by modification of lifestyle? ( )% |
| --- |
